# The Role of Neutrophil to Lymphocytes Ratio (NLR) as a Predictor of Disease Activity in Behcet’s Syndrome—A Comprehensive Review

**DOI:** 10.3390/jcm14165847

**Published:** 2025-08-19

**Authors:** Rula Daood, Firas Sabbah, Abdallah Fawaz, Fadi Hassan, Mohammad E. Naffaa

**Affiliations:** 1 Rheumatology Unit, Galilee Medical Center, Nahariya 2210011, Israel; rulad@gmc.gov.il (R.D.);; 2 The Azrieli’s Faculty of Medicine, Bar-Ilan University, Safed 5290002, Israel; 3 Rheumatology Unit, HaTsafon Medical Center, Poriya 1520801, Israel; 4 Rheumatology Unit, Ha’Emek Medical Center, Afula 1834111, Israel; 5 Rappaport’s Faculty of Medicine, Technion, Haifa 3200003, Israel

**Keywords:** neutrophil-to-lymphocyte ratio (NLR), Behçet’s syndrome (BS)

## Abstract

Behçet’s syndrome (BS) is a chronic, relapsing inflammatory disease with multisystem involvement and prominent neutrophil activation. The neutrophil-to-lymphocyte ratio (NLR) has gained increasing attention as a potential surrogate marker for systemic inflammation. In this review we aimed to summarize and critically review the current evidence regarding the utility of NLR in BS, including its association with overall disease activity and specific organ involvement, as well as to explore its strengths and limitations as a clinical biomarker. NLR seems to be a simple, accessible, and cost-effective biomarker that can be elevated in Behçet’s syndrome and tends to be higher during active disease. Studies have demonstrated its consistent correlation with overall disease activity, as well as with specific manifestations such as mucocutaneous, ocular, vascular, and articular involvement. Moreover, NLR levels have been shown to decrease in response to anti-inflammatory treatments, supporting its potential utility in monitoring treatment effectiveness. Despite its seemingly cost-effective features, its routine integration into daily practice remains largely limited, mainly due to low specificity and the lack of standardized cut-off level. Further prospective studies are needed to assess its use in daily practice before it can be integrated into any disease activity score.

## 1. Introduction

Behcet’s syndrome (BS) is a chronic, multisystem, inflammatory condition with a relapsing and remitting course. Recurrent oral and genital ulceration, the hallmark of BS, are the most common clinical manifestations, but other organs can be involved, including, but not limited to, eyes, gastrointestinal tract, central nervous system and vascular tree. BS is particularly prevalent along the ancient “Silk Road,” especially in Turkey, the Middle East, and Southeast Asia. The etiology of BS remains to be elucidated but it is believed to involve the complex interplay between genetic predisposition (e.g., HLA-B51), environmental triggers, and abnormal immune responses [1].

Neutrophils play a central role in the pathogenesis of BS, as suggested by several observations, including the activation of Th17 cells and endothelium, secretion of neutrophil extracellular traps (NETs) and reactive oxygen species (ROS) and the presence of neutrophils in several typical lesions of BS, such as oral ulcers, pathergy and the GI tract [2,3].

Currently, no single, specific biomarker can reliably assess disease activity. Therefore, assessment of disease activity is based mainly on recent medical history, as exemplified in the Behçet’s Disease Current Activity Form (BDCAF) or the Physician Global Assessment, alongside non-specific inflammatory markers like C-reactive protein (CRP) and erythrocyte sedimentation rate (ESR) [4]. In parallel, growing attention has been directed towards simple and accessible inflammatory markers derived from routine blood tests. Among these, the neutrophil-to-lymphocyte ratio (NLR), calculated by dividing the absolute neutrophil count by the absolute lymphocyte count, has gained interest as reflecting the balance between the innate (neutrophils) and adaptive (lymphocytes) immune responses [5]. Cumulative evidence supports the use of NLR as a cost effective and simple disease activity marker for endothelial activation and inflammation in many diseases, including BS. NLR has been studied in a wide range of clinical conditions, including ischemic stroke, cerebral hemorrhage, cardiovascular events, sepsis, infectious diseases, and cancer and has been found to be associated with increased disease burden and poor clinical outcomes [6,7]. In recent years, growing attention has been directed towards the utility of NLR in autoimmune diseases. Elevated NLR values have been reported in psoriasis, ulcerative colitis, rheumatoid arthritis, systemic lupus erythematosus, primary Sjögren’s syndrome, Takayasu’s arteritis, and BS, suggesting its potential use as a surrogate marker for systemic inflammation and disease activity [8,9,10,11,12]. There is a growing interest in NLR as a potential inflammatory biomarker in BS, mainly due the central role of neutrophils in the pathogenesis of BS. Several studies have explored its utility, suggesting a possible correlation between elevated NLR values and active disease. The first study that examined the role of NLR in BS was reported by Rifaioglu et al. in 2014 [13]. They demonstrated that NLR levels were higher in patients with active BS compared with both healthy controls and patients with inactive disease [13]. In a recent large-scale retrospective study, Mentesoğlu et al. (2024) [14] analyzed 513 BS patients and showed that mean NLR values were significantly higher among patients with active disease compared with those with inactive disease (3.50 vs. 1.88; *p* < 0.001). Elevated NLR levels were also associated with various clinical presentations, including mucocutaneous, vascular, neurological, and articular involvement [14].

Studies have investigated the performance of NLR in a variety of clinical contexts in BS, including its correlation with overall disease activity, specific organ involvement, and its potential role in predicting disease course or treatment response.

This review aims to comprehensively review the current literature regarding the role of NLR in BS, including its correlation with acceptable and validated indices of disease activity, its performance as a measure of global disease activity and specific organ activity, and its performance as a predictor of treatment response and future complications.

## 2. Methods

We conducted a comprehensive literature search from January 2014 through June 2025 through MEDLINE (via PubMed) and Google Scholar for studies reporting NLR in BS patients. Searches combined MeSH terms and keywords—“Behçet’s syndrome,” “neutrophil-to-lymphocyte ratio,” and related synonyms—using Boolean operators (AND/OR). The reference lists of the retrieved articles were hand searched to identify additional relevant publications. Studies were eligible if they met the following criteria: (1) they were case–control, cross-sectional, or cohort studies; (2) they provided information on NLR in BS and controls; and (3) no restrictions on language or ethnicity were applied.

Exclusion criteria comprised duplicate publications or studies with insufficient data. From each included article, we extracted the first author, publication year, sample size, and reported NLR values (mean ± SD and/or median [IQR]); additionally, we collected clinical parameters such as NLR in active versus inactive disease and its correlation with specific manifestations of BS, differences between NLR level in BS vs. controls, NLR level in active vs. inactive disease and correlation with other inflammatory markers.

## 3. Role of Neutrophils in Behçet’s Syndrome

Neutrophils, as a key component of the innate immune system, have long been implicated in the pathogenesis of BS, with early studies dating back to the 1970s demonstrating enhanced chemotactic activity in neutrophils from BS patients. Histopathological examinations of various tissues, including mucosal, aortic, intestinal, conjunctival, and cutaneous lesions, have shown prominent neutrophilic infiltration, particularly around the vasa vasorum [2]. Similar patterns of infiltration have been documented in neurological involvement (neuro-Behçet’s) and in erythema nodosum-like lesions [2]. The mechanisms driving increased neutrophil activity in BS are multifactorial and may involve soluble mediators, microbial products, and direct cellular interactions. Upregulation of adhesion molecules on both neutrophils and endothelial cells is thought to facilitate recruitment and transmigration [2]. Supporting these histological and pathophysiological findings, flow cytometry studies have demonstrated the upregulation of activation markers such as CD64, CD11a, CD11b, CD18, CD206, CD209, and CD66b on neutrophils from BS patients, indicating a hyper-activated neutrophil profile. In parallel, increased levels of neutrophil granule enzymes—including neutrophil elastase, myeloperoxidase (MPO), and S100A proteins—have been reported in the saliva and serum, reflecting systemic neutrophil activation in BS [2]. Additionally, sera from BS patients have been shown to enhance the adhesion of healthy neutrophils to human endothelial cells, alongside elevated levels of CXCL-8, a potent neutrophil chemoattractant [15].

In recent years, increasing attention has been directed to the role of NETs in the pathogenesis of BS. Neutrophils contribute to disease mechanisms through multiple effector functions, including phagocytosis, degranulation, and NET formation. NETs are web-like structures composed of chromatin fibers decorated with histones and neutrophil-derived enzymes, released through a distinct form of cell death known as NETosis [16]. Beyond their antimicrobial functions, NETs have been implicated in the pathogenesis of several autoinflammatory and autoimmune diseases, including systemic vasculitides. In BS, they are increasingly recognized as key contributors to endothelial damage and thrombus initiation and propagation [2]. NETosis can be triggered by several stimuli, including pathogens, proinflammatory cytokines, pathogen-associated molecular patterns (PAMPs), damage-associated molecular patterns (DAMPs) and immune complexes [16]. One of the major molecular pathways leading to NETosis is the generation of ROS. ROS are the products of biological reduction reactions. Uncontrolled free radicals in the living organism cause disease and cell damage [17]. NETosis is mediated by NADPH oxidase, resulting in the formation of superoxide anions (O_2_^−^) and hydrogen peroxide (H_2_O_2_), which are further processed by MPO into highly reactive secondary oxidants. While ROS are crucial for pathogen clearance, under certain circumstances, such as during autoimmune response, neutrophils can be triggered to release ROS and proteases extracellularly causing damage to host tissues, modification of host proteins, lipids and DNA and dysregulation of oxidative homeostasis. Studies have consistently shown elevated ROS levels and reduced antioxidant defenses in both the plasma and neutrophils of BS patients. Moreover, excessive ROS may contribute to fibrinogen oxidation, resulting in delayed fibrin polymerization and resistance to fibrinolysis—mechanisms that may underlie the thrombus formation process frequently observed in BS [18]. Supporting these findings, Becatti et al. have demonstrated that neutrophils from patients with BS exhibit significantly elevated ROS production. This enhanced oxidative stress leads to fibrinogen carbonylation and alterations in its secondary structure, resulting in impaired clot formation and decreased susceptibility of fibrin to plasmin-induced lysis. These suggested mechanisms may further contribute to thrombus formation in patients with BS [19]. ROS generation, together with calcium mobilization, triggers a cascade of intracellular events that culminates in NETosis. These include nuclear delobulation, enzymatic activation of MPO and NE, and chromatin modification via histone citrullination by peptidyl arginine deiminase 4 [2].

Le Joncour et al. analyzed circulating cell-free DNA and MPO–DNA complexes—key NETosis biomarkers—in patients with BS. Both markers were significantly elevated in active disease compared with inactive patients and healthy controls. The highest levels were observed in patients with vascular involvement. Furthermore, purified neutrophils from BS patients exhibited spontaneous NETosis, in contrast to healthy controls. These findings support the hypothesis that enhanced NET formation contributes to the thrombotic and vasculopathic features of BS [20].

Accumulating evidence suggests that IL-17-producing Th17 cells play an important role in the pathogenesis of BS. These cells contribute to inflammation by secreting proinflammatory cytokines and by promoting neutrophil recruitment via the granulocyte colony-stimulating factor pathway [3]. Patients with active BS exhibit increased proportion of circulating Th17 cells and enhanced IL-17 production by peripheral blood mononuclear cells. IL-21, a promoter of Th17 responses, has been shown to contribute to the expansion of Th17 cells in the peripheral blood of patients with BS, while concurrently reducing the number of FoxP3-expressing regulatory T cells (Tregs). Altogether, the interplay between Th17 cells and neutrophils may sustain a self-amplifying inflammatory loop that bridges innate and adaptive immunity in BS [3].

From a therapeutic perspective, several agents used in the management of BS have been shown to attenuate neutrophil activation and reduce the formation of NETs. Colchicine, as demonstrated in the study by Korkmaz et al., modulates neutrophil activity by lowering intracellular calcium concentrations (Ca^2+^), a key trigger of ROS production. By suppressing Ca^2+^ influx, colchicine reduces oxidative stress and limits neutrophil-mediated tissue damage in BS [17]. Similarly, glucocorticoids, anti-TNFα therapies, and apremilast have also been reported to inhibit neutrophil activation and NET release [2].

**Figure 1:**
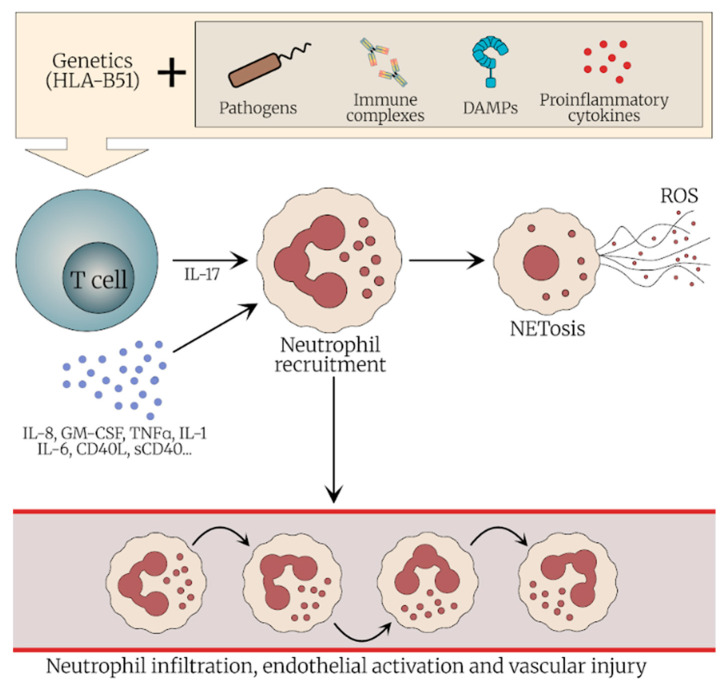
The role of neutrophils in the immunopathogenesis of Behçet’s syndrome. Neutrophils play a key role in the pathogenesis of Behçet’s syndrome through cytokine-mediated and IL-17-driven activation, leading to enhanced tissue infiltration. Activated neutrophils release ROS and promote NETosis, causing endothelial activation, sustained inflammation, and thrombus formation. Neutrophils have been detected in multiple tissues, including the skin, mucosa, vasculature, and gastrointestinal tract. Abbreviations: DAMPS—damage-associated molecular patterns, ROS—reactive oxygen species, GM-CSF—granulocyte-macrophage colony-stimulating factor.

Figure 1 describes the potential role of neutrophils in BS, highlighting their activation by various cytokines, including IL-17, production of ROS, NETosis, and infiltration into affected tissues.

## 4. Physiological Background of NLR and Normal Limits

NLR reflects, grossly, the balance between innate and adaptive immune responses and serves as a reliable indicator of both inflammation and stress. The reciprocal changes in neutrophil and lymphocyte counts represent a complex, multifactorial process influenced by regulation of various immunologic, neuroendocrine, humoral, and biological mechanisms [5]. Although the separate role of neutrophil and lymphocyte counts in the clinical severity of systemic inflammatory response has been previously examined, the concept of NLR was initially proposed by Zahorec in 2001, as a simple marker of systemic inflammatory response and physiological stress in critically ill patients, particularly when assessing the severity of sepsis and systemic infections, including bacteremia [21]. This parameter, which is formulated by the ratio of two simple complete blood count parameters, has been found to provide better performance in the prognosis of many critical conditions than its components [22]. An increase in NLR is determined by an increase of neutrophils and/or reduction in lymphocytes. Elevated neutrophil counts and the corresponding rise in the NLR are common findings in a variety of clinical settings associated with acute or chronic inflammation. These include bacterial and fungal infectious diseases, acute cerebrovascular events, myocardial infarction, atherosclerotic processes, malignancies, major trauma, and complications following surgery. Such elevations often reflect the early proinflammatory phase of systemic responses, where neutrophils are thought to play central role [23]. In the context of systemic inflammatory response syndrome, the inhibition of neutrophil apoptosis further amplifies their activity, contributing to increased NLR and typically characterized by both neutrophilia and relative lymphopenia [23].

In contrast, lymphocytes play a central role in the adaptive immune system, enabling the targeted elimination of pathogens, infected cells, and even malignant or premalignant cells. A reduction in circulating lymphocyte counts can be observed following acute infections, use of immunosuppressive or cytotoxic therapies (e.g., chemotherapy), and in various immune-mediated diseases such as systemic lupus erythematosus [6]. The mechanisms responsible for lymphopenia also involve margination and redistribution of lymphocytes within the lymphatic system, along with accelerated apoptosis through tumor-related cytokines, particularly interleukin-10 (IL-10) and tumor necrosis factor beta [22].

Beyond its diagnostic implications, NLR has also been associated with prognostic value in the general population. Higher NLR levels have been linked to increased overall mortality and specific mortality risks, including cardiovascular diseases, chronic respiratory conditions, infections such as pneumonia and influenza, and renal disorders, with hazard ratios rising progressively across NLR quartiles [6,23]. Notably, NLR association with overall mortality appeared strongest in the immediate 1-year period after the baseline blood measurement [6].

NLR values can be influenced by various physiological and demographic factors, including age, sex, smoking status, obesity, diabetes mellitus, and ethnicity, and lower values have been reported among African American individuals [5,24].

Corticosteroids can alter the NLR values by reducing peripheral lymphocyte counts and increasing granulocyte counts, thereby elevating NLR. In a randomized trial of low-dose corticosteroids in men with castration-resistant prostate cancer, those receiving prednisolone experienced a significant rise in NLR—from a baseline median of 2.6 to 3.7 at six weeks to 4.1 at twelve weeks (both *p* < 0.001). This increase was driven primarily by neutrophil expansion, with stable lymphocyte counts [25].

Several epidemiological studies have evaluated normal NLR values in healthy populations, showing relatively high variability by ethnicity and geography. However, the average NLR is generally around 1.65, with commonly accepted normal values ranging between 1.2 and 2.15 [5,22]. Although earlier studies have proposed broader upper limits of up to 5, distinct cutoff values have been reported for different diseases (e.g., malignancy, sepsis, and cardiovascular diseases), and there remains a lack of consensus as to the unified pathological value in this regard [22]. Values higher than 3 and below 0.7 in adults are generally pathological. An NLR between 2 and 3 is considered within the grey zone corresponding to latent, subclinical or low-grade inflammation [5]. Several population-based studies have examined these variations. Azab et al. have reported a mean NLR of 2.15 in large US cohort, while Lee et al. found a lower average of 1.65 in the Korean population [24,26]. Overall, higher NLR values are generally associated with more severe inflammation and physiological stress [5].

## 5. NLR in Behçet’s Syndrome

Cumulative evidence supports the role of the NLR as a simple, accessible, and cost-effective biomarker for systemic inflammation in BS. Numerous studies have investigated its association with disease activity and clinical manifestations. Rifaioglu et al. have reported that NLR was found to be higher in 65 BS patients versus 100 controls, and markedly elevated in active disease, correlating with CRP and white blood cell counts [13]. Similarly, a case-control study by Selim et al. [27], including 36 BS patients and 36 healthy controls, demonstrated significantly elevated levels of both NLR and platelet-to-lymphocyte ratio (PLR) in BS patients (*p* = 0.008 and 0.011, respectively). The median NLR in the BS group was 3.5 (0.4–9.6), compared with 1.7 (0.8–9.8) in the control group (*p* = 0.008). Supporting these findings, a recent meta-analysis by Lee et al. reported significantly higher NLR values in BS patients compared with healthy controls, with a standardized mean difference of 1.312 (95% CI: 0.713–1.911; *p* < 0.001), indicating a large effect size [28].

In the context of active BS, NLR has emerged as a potential marker of inflammatory activity. The same cohort reported by Selim et al. [27] showed that active BS patients had significantly higher NLR than those with inactive disease (median NLR: 3.8 [1.0–5.4] vs. 1.8 [0.4–9.6]; *p* = 0.009). Additional inflammatory markers—including PLR, lymphocyte-to-monocyte ratio (LMR), ESR, and CRP—also differed significantly between active and inactive groups (*p* < 0.05), with active patients showing elevated NLR, PLR, ESR, and CRP, and reduced LMR. More recently, Mentesoglu et al. have analyzed a large retrospective cohort of 513 adult BS patients to explore the association between inflammatory indices—including NLR—and disease activity. Receiver operating characteristic (ROC) curve analysis revealed an optimal NLR cutoff value of 2.11, yielding fair sensitivity and specificity rates of 67.3% and 68.4% for identifying active disease, respectively (AUC = 0.750). The mean NLR was significantly higher among patients with active BS compared with those with inactive BS (3.50 vs. 1.88; *p* < 0.001) [14]. Balkarli et al. [29], Hammad et al. [30], Djaballah-Ider et al. [31], and Shadmanfar et al. [32] have all reinforced this association, collectively underscoring the utility of NLR as a surrogate marker for disease activity in BS.

Several of these studies have also demonstrated associations between elevated NLR and specific clinical manifestations, including mucocutaneous and articular involvement [14,27,30,31], gastrointestinal manifestations [30], vascular involvement [14,31], neurologic symptoms [14,27], ocular involvement [27,32] and increased carotid intima-media thickness [33]. Supporting the ocular relevance of NLR, a hospital-based study by Avci et al. involving 912 BS patients showed that NLR, along with PLR and mean platelet volume (MPV), were all significantly elevated in patients with anterior uveitis compared with BS patients without ocular involvement and healthy controls. Among the three parameters, NLR demonstrated the strongest diagnostic performance, suggesting its utility in identifying anterior uveal inflammation in BS [34]. In a study involving 114 patients diagnosed with Behçet uveitis, the optimal cutoff value for the NLR associated with poor visual outcomes was identified as 5.608 [35].

Djaballah-Ider et al. further showed that NLR is treatment-responsive, as the NLR value declined after 15 days of combined colchicine and corticosteroid therapy in active BS patients. They have also reported that NLR values were found to be highest in naive active patients with mucocutaneous or angio-Behçet manifestations, exceeding those seen in ocular or articular disease [31]. Lee et al. demonstrated that NLR is particularly elevated during active disease phases, reinforcing its role as a reliable and reproducible marker for monitoring disease activity [28]. Zhang et al. proposed combining NLR with hemoglobin levels to enhance diagnostic accuracy [36].

Taken together, these findings underscore NLR’s clinical utility in BS as a biomarker reflecting systemic inflammation, disease activity, and specific organ involvement. Its consistency across multiple studies and populations supports its use in clinical practice and research settings. Nevertheless, it should be emphasized that NLR has not been adapted by clinicians in daily practice when treating patients with BS. This is likely due to lack of specificity, as well as the lack of standardized cutoff values, insufficient prospective validation, and the absence of clear clinical consensus regarding its implementation.

**Figure 2:**
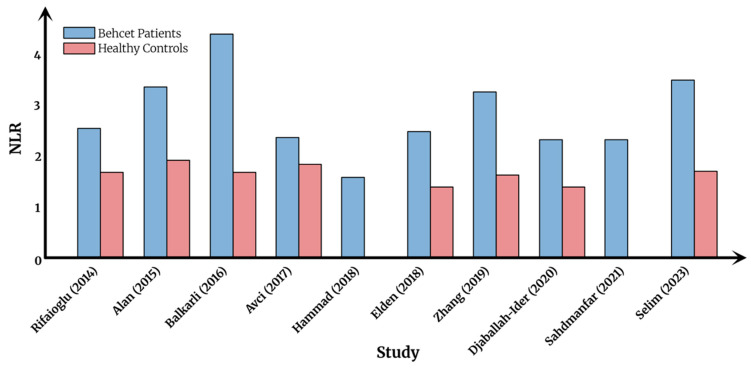
NLR levels in Behcet syndrome vs. healthy controls.

Table 1 summarizes the key studies evaluating NLR in patients with BS. Figure 2 provides a visual comparison of NLR values reported in BS patients versus healthy controls across selected studies, highlighting the wide variations across different studies.

## 6. Additional Neutrophil-Related Indices in Behçet’s Syndrome

The Systemic Immune–Inflammation Index (SII), introduced in 2014 and derived from the NLR, is calculated as (platelets × neutrophils)/lymphocytes. By incorporating platelet count, the SII offers a broader reflection of systemic inflammation and has shown prognostic value in cancers, cardiovascular diseases, and infections [22]. Tanacan et al. [38] have found an elevated SII and absolute neutrophil count (ANC) in patients with active BS, suggesting that SII values above 552 × 10^3^/mm^3^ may help to identify active disease. Similarly, Mentesoglu et al. have demonstrated that SII is significantly higher in active BS, with a proposed cutoff of 526.23 × 10^3^/mm^3^, showing 70.4% sensitivity and 70.3% specificity [14].

The Systemic Inflammation Response Index (SIRI), which is calculated by the formula = neutrophil × monocyte/lymphocyte, was introduced in 2016. Initially proposed as a prognostic marker in malignancy, SIRI has also been explored in various diseases characterized by prominent inflammatory responses, with growing interest in its potential diagnostic and prognostic utility across diverse clinical settings [22].

A more recent inflammatory marker, the Pan-Immune–Inflammation Value (PIV), is calculated as follows: neutrophil count × platelet count × monocyte count/lymphocyte count (all expressed in 10^9^/L). The PIV was recently investigated in the context of vascular Behçet’s syndrome (VBS), where the cutoff value of ≥261.6 was identified as an independent predictor of VBS [odds ratio (OR): 2.758; 95%; confidence interval (CI): 1.327–5.736; *p* = 0.007] [39].

## 7. Strengths and Limitations in NLR Use in Rheumatic Diseases Including Behçet’s Syndrome

In BS, where specific and reliable biomarkers for diagnosis and disease monitoring are lacking, the NLR offers several notable advantages. Unlike clinical symptoms, which may be subjective and influenced by patient perception, NLR provides an objective and quantifiable indicator of systemic inflammation. It is non-invasive, inexpensive, widely accessible and easily derived from routine blood tests without requiring specialized assays. NLR reflects the underlying immune dysregulation central to BS pathogenesis by incorporating both neutrophilia and relative lymphopenia. Several studies have shown significant correlation between elevated NLR and disease activity in BS, including specific manifestations such as vascular involvement and ocular flares. NLR may serve as a valuable adjunct to clinical evaluation, particularly in resource-limited settings, by supporting flare assessment and treatment decisions. Its integration into routine assessment could enhance the precision of disease activity evaluation and complement clinical judgment.

Despite its advantages, NLR presents several limitations. The first and foremost is its lack of specificity. Elevated NLR values may be observed in a wide range of non-rheumatic conditions, including infections, malignancies, psychological stress, and metabolic disorders [6,7]. Second, while NLR has shown consistent correlation with mucocutaneous and articular involvement in BS, its utility may be limited in assessing other disease manifestations. Third, the absence of standardized cutoff values limit its uniform application in clinical practice [5,22]. Fourth, numerous confounding factors, including medication use, age, sex, smoking status, obesity, diabetes mellitus, and ethnicity, influence NLR values and hinder their interpretability [24,31]. Fifth, broader hematologic profiling suggests that, while NLR and PLR tend to rise with BS activity, other indices such as the lymphocyte-to-monocyte ratio and MPV may display inverse or non-linear trends, supporting the need for a multimodal approach to disease monitoring [27].

Another key limitation of using NLR is its implicit assumption that all circulating neutrophils constitute a single, uniform population. Advances in single-cell technologies and functional assays have unveiled distinct neutrophil subsets with diverse phenotypes and functional capabilities, challenging the notion of neutrophils as a homogeneous population. Neutrophil heterogeneity manifests across multiple dimensions—maturation state, density, surface marker expression, and functional polarization—and specialized subsets such as tumor-associated neutrophils and low-density granulocytes exhibit unique cytokine profiles, tissue-homing capacities, and roles in disease pathogenesis [40]. Because NLR captures only total neutrophil counts, it cannot discriminate between these functionally and phenotypically diverse subpopulations, which may blunt its specificity and obscure subtle changes in the immune response.

Several key considerations must be addressed for the effective clinical application of NLR, including sufficient time for proper contextual interpretation. Ideally, NLR should be measured at multiple time points, as tracking its dynamic fluctuations may enhance clinical relevance. It has been proposed that calculating an average NLR across different stages of disease, rather than relying on a single-point measurement, can yield more reliable insights. Similarly, the use of delta (∆) NLR—reflecting changes from baseline—may improve its predictive and prognostic value. However, the optimal method for NLR assessment, whether through serial monitoring, baseline comparison, or averaging, remains to be defined.

Therefore, large-scale prospective studies are still needed to validate its clinical utility, establish standardized cutoff values, and determine its precise role in various clinical contexts. Until such evidence is available and consensus is reached, we believe that NLR cannot be adopted for routine use in daily clinical practice.

In our dedicated BS clinic daily practice, we incorporate NLR measurements as an adjunct to comprehensive disease assessment rather than a standalone measure. We apply this metric specifically in steroid-free patients, thereby avoiding steroid-induced confounding. We use serial NLR measurements alongside clinical evaluation, standard laboratory markers (CRP, ESR) and disease activity forms (BDCAF) for longitudinal monitoring. It is essential to interpret NLR values within their clinical context and after ruling out other non-Behçet-related causes of elevated NLR, that may spuriously increase the ratio.

**Figure 3:**
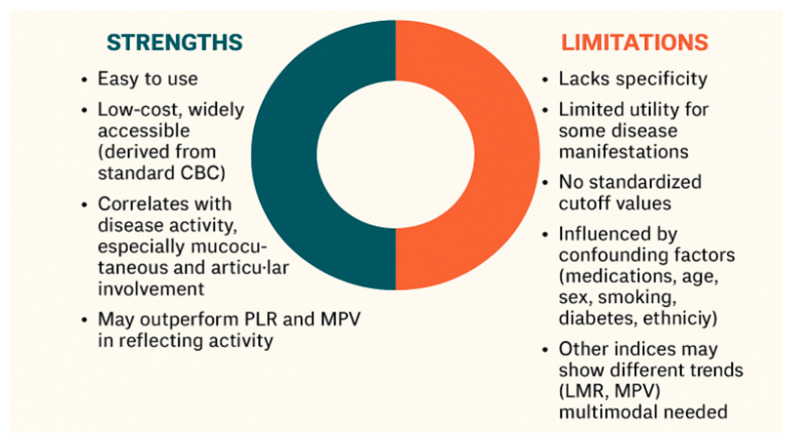
Strengths and limitations associated with the use of NLR in Behçet’s syndrome. Abbreviations: CBC—complete blood count, PLR—platelet-to-lymphocyte ratio, MPV—mean platelet volume, LMR—lymphocyte-to-monocyte ratio.

Figure 3 illustrates the key strengths and limitations of using NLR as a biomarker in BS.

## 8. Future Research Directions

Several key directions may enhance the clinical utility and research applications of NLR in BS. Establishing standardized cutoff values is essential, taking into consideration organ-specific involvement and individual factors such as age, sex, and ethnicity. Prospective multicenter studies are needed to validate its diagnostic and prognostic validity across other BS populations.

Integrating NLR into composite disease activity indices or predictive models may further improve its relevance in clinical decision-making. Additionally, longitudinal studies assessing NLR dynamics over time in response to treatment could provide valuable insights into disease monitoring and flare prediction.

## 9. Conclusions

NLR is a simple, accessible, and cost-effective biomarker that has shown consistent correlation with disease activity in BS, particularly in cases with mucocutaneous, articular, ocular, and vascular involvement, and can also act as an indicator of treatment response. It may work as a valuable adjunct to clinical evaluation and to enhance the ability to track systemic inflammation and help tailor treatment for specific patients. NLR has the potential to serve as a prognostic marker for disease severity and even mortality, provided that relevant potential confounding factors are identified and taken into account. Nevertheless, several limitations—including the lack of specificity, influence of confounding factors, and absence of standardized cutoff values—currently preclude the routine use of NLR in clinical practice. Its variability across disease stages and organ involvement necessitates further refinement in measurement strategy.

Future prospective, multicenter studies are essential to validate its clinical utility, define standardized thresholds, and determine the best way to integrate NLR into disease activity indices. Until then, NLR should be considered a valuable adjunctive tool—particularly in resource-limited settings—but not a standalone marker for decision-making in BS.

## Figures and Tables

**Table 1:** Studies evaluating neutrophil-to-lymphocyte ratio in Behçet’s syndrome.

	Author Name	Year	Study Type	BS Patients (n)	Controls (n)	NLR in BS	NLR IN Controls	Format	Statistical Significance
1	Rifaioglu [13]	2014	Retrospective (cross-sectional) study	65	100	2.55 ± 1.57	1.7 ± 0.8	Mean ± SD	*p* < 0.001
2	Alan [37]	2015	Retrospective (cross-sectional) study	254	173	3.37 ± 2.74	1.95 ± 1.21	Mean ± SD	*p* < 0.001
3	Balkarli [29]	2016	Retrospective (cross-sectional) study	120 active/ 66 inactive	79	4.41 (2.77–6.20) active 1.82 (1.47–1.99) inactive	1.72 (1.32–2.04)	Median (IQR)	*p* < 0.001 ††
4	Avci [34]	2017	Retrospective (cross-sectional) study	71 with uveitis/69 without uveitis	151	2.37 (0.96–6.36) with uveitis 1.54 (1.38–1.81) without uveitis	1.88 (1.52–2.26)	Median (IQR)	*p* < 0.001
5	Hammad [30]	2018	Retrospective (cross-sectional) study	16 active/7 inactive	-	1.59 ± 0.13 active, 1.447 ± 0.093 inactive	-	-	*p* = 0.016
6	Elden [33]	2018	Retrospective (cross-sectional) study	20	20	2.5 ± 1.3	1.42 ± 1.8	Mean ± SD	*p* <0.05
7	Zhang [36]	2019	Retrospective (cross-sectional) study	67	92	3.27 (1.85∼4.77)	1.66 (1.3∼1.91)	Median (IQR)	*p* < 0.001
8	Djaballah-Ider [31]	2020	Retrospective (cross-sectional) study	47 active (naive/treated *)/14 inactive	25	2.3 ± 0.30 naive, 1.8 ± 0.25 treated 1.36 ± 0.21 inactive **	≈1.4 ± 0.2 **	Mean ± SD	*p* < 0.0001 †
9	Shadmanfar [32]	2021	Retrospective (cross-sectional) study	319	-	2.34 ± 1.88	-		
10	Selim [27]	2023	Retrospective (case-control) study	36	36	3.5 (0.4–9.6)	1.7 (0.8–9.8)	Median (IQR)	0.008
11	Dilek Mentesoglu [14]	2024	Retrospective (observational) study	533 active/158 inactive	-	3.50 active, 1.88 inactive	-		<0.001

†† In all groups. * Treatment with colchicine and corticosteroids. ** These are approximate values obtained by visually extracting data from the article’s figure. † This *p*-value refers specifically to the comparison of NLR between the naïve active BS group before therapy and the same patients after 15 days of combined colchicine + corticosteroid treatment. Abbreviations: BS—Behcet syndrome, NLR—neutrophil-to-lymphocyte ratio.
